# Three-photon excited fluorescence imaging in neuroscience: From principles to applications

**DOI:** 10.3389/fnins.2023.1085682

**Published:** 2023-02-20

**Authors:** Yujie Xiao, Peng Deng, Yaoguang Zhao, Shasha Yang, Bo Li

**Affiliations:** State Key Laboratory of Medical Neurobiology, Department of Neurology, Ministry of Education (MOE), Frontiers Center for Brain Science, Institute for Translational Brain Research, Huashan Hospital, Fudan University, Shanghai, China

**Keywords:** three-photon excited fluorescence microscopy, deep brain imaging, structural imaging, functional imaging, rodents

## Abstract

The development of three-photon microscopy (3PM) has greatly expanded the capability of imaging deep within biological tissues, enabling neuroscientists to visualize the structure and activity of neuronal populations with greater depth than two-photon imaging. In this review, we outline the history and physical principles of 3PM technology. We cover the current techniques for improving the performance of 3PM. Furthermore, we summarize the imaging applications of 3PM for various brain regions and species. Finally, we discuss the future of 3PM applications for neuroscience.

## 1. Introduction

**FIGURE 1:**
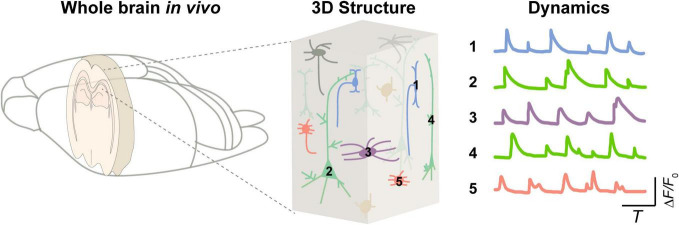
Integrated brain dynamics in a 3D structure. **(Left)** A rodent brain. **(Middle)** 3D architecture inside a specific brain region from superficial to deep layers. **(Right)** Activity of various types of cells.

One of the vital goals in neuroscience is to elucidate the diverse structures and functions of intricate neural circuits (Klausberger and Somogyi, 2008; Shenoy et al., 2013; Luo, 2021). But, today, it remains unclear how various neuronal components integrate into a whole brain to carry out logical operations (as shown in Figure 1). Observing the dynamics of various components in the brain is essential for decoding the principles underlying brain physiological functions (Sajad et al., 2019; Yildirim et al., 2019; McColgan et al., 2020; Peters et al., 2021) and for understanding pathological conditions (McGregor and Nelson, 2019; Maestú et al., 2021). Exploring the neural activity inside the brain is still a challenge. Such needs propel the development of advanced neurotechniques (Wilt et al., 2009; Yoon et al., 2020; Choquet et al., 2021).

The nervous system is featured with high degree of structural and functional complexities, largely because the brain tissue is interwoven with neurons, glia, blood vessels and so on (Paredes et al., 2018; Kugler et al., 2021), and brain functions could be driven by electrical, chemical, and mechanical signals occurring in the nervous systems. Our capability to understand the complexity of the nervous system is limited by the capability of tools to probe neural signaling in such a complex system (Russell et al., 2022). Although emerging methods (particularly used in the mouse brain) based on electronic (Mizuma et al., 2010; Kodandaramaiah et al., 2012), ultrasonic (Rabut et al., 2019, 2020), and magnetic (Tsurugizawa et al., 2020) signals offer application-specific advantages for understanding how information is transferred and processed within the mammalian nervous system, these approaches remain insufficient to dissect the principles of neural communication across the diverse range of their signaling modalities. For example, conventional electronics, such as patch clamp (Davie et al., 2006) and electroencephalography (EEG) (Drinkenburg et al., 2016) recordings, do not recapitulate the chemical and mechanical properties of neural tissue, and they are also inaccessible for investigating the dynamics of the electrically inactive glia. Solely electrophysiological approaches suffer from the fact that recordings are invasive and/or unable to reconcile single-cell resolution with high-throughput. The spatiotemporal resolution limitation is challenging to overcome for neuroimaging methods such as positron emission tomography (PET), functional magnetic resonance imaging (fMRI), and ultrasound imaging, despite their ability to acquire high-throughput data. Optics can overcome the aforementioned limitations in specific situations. As a result, imaging techniques based on optics have been widely used in neuroscience.

Optical imaging approaches offer wide ranges of spatial and temporal resolutions and the accessibility of various physiological components in the brain. Optical imaging methods typically used for neuroscience include fiber photometry (Li et al., 2019), miniscope with grade index (GRIN) lens (Resendez et al., 2016), light-sheet microscope (Olarte et al., 2018; Voigt et al., 2019; Stelzer et al., 2021), two-photon microscope (2PM) and three-photon microscope (3PM) (Lecoq et al., 2019; Wang and Xu, 2020). A merit of optical imaging approaches is that they allow us to visualize neuronal structures and activities at multiple spatial and temporal scales, from individual fine structures (such as spines and axonal boutons) to whole brain networks, and from milliseconds to multiple months. When combined with cutting-edge optical tools, particularly various genetically encoded fluorescent indicators (Chen et al., 2013; Knöpfel and Song, 2019; Sabatini and Tian, 2020; Kim and Schnitzer, 2022), optical imaging enables us directly to label specific types of neurons and to monitor their activities in the nervous system with high spatiotemporal resolution. Optical imaging methods, especially fluorescence microscopies, have expanded the frontiers of neuroscience research and helped us uncover details of neural coding that are hard to be figured out with conventional methods.

Fluorescence microscopies can be classified into single-photon, two-photon (2P) and three-photon (3P) microscopies, considering their differences in fluorescence excitation mechanisms. The merits of fluorescence microscopies are easy to see. They can be used to simultaneously image multiple cell types with great sensitivity and high throughput either *in vivo* or *in vitro*. Conventional fluorescence microscopies are designed based on single-photon fluorescence excitation, in which the fluorophore absorbs one excitation photon and emits one fluorescence photon. In general, the imaging depth is limited to tens of micrometers because of the strong out-of-focus background fluorescence signal. It is therefore challenging to use conventional fluorescence microscopy for *in vivo* deep imaging of nervous systems. However, 2PM and 3PM are designed based on 2P/3P excitation, in which the fluorophore absorbs two or three photons almost simultaneously and emits one fluorescence photon. The natural advantage of 2P/3P excitation brings possibilities to largely reduce out-of-focus background fluorescence and increase imaging depth.

The advent of 2PM has partially alleviated the challenge of insufficient imaging depth to some extent (Helmchen and Denk, 2005). 2PM has two key characteristics. Firstly, the optical section (or suppression of the out-of-focus background fluorescence) can be obtained by combining the non-linear fluorescent excitation process with the convergence of light by a microscopy objective. The focus has higher optical intensity, and thus it excites much more fluorescent signal than the out-of-focus area, since the fluorescent signal is proportional to the square of optical intensity. Secondly, the wavelength of 2P excitation is usually around twice that of one-photon excitation (1PE), and the longer the wavelength is, the less it scatters in biological tissues. This results in an increase in the penetration depth of 2PM, so the focus can reach the deeper tissue in the brain. The two features make 2PM suitable for deep imaging in living organisms. In the past 30 years, 2PM has brought the opportunity for novel understanding of mouse brain functions *in vivo* (Lecoq et al., 2019; Villette et al., 2019; Wu et al., 2020; Zong et al., 2021) and has been widely used in neuroscience research, especially in the cerebral cortex. However, 2P imaging has also shown its limits for deeper brain tissue, such as the hippocampus. The imaging depth of a standard 2PM is limited to ∼600 μm. For deeper exploration, the scattering of light is so strong that only a very small fraction of the excitation light can reach the focal point and cannot excite enough fluorescent signal. In addition, most of the scattered excitation light excites stronger out-of-focus background fluorescence than the focus, which blurs the image and lowers the signal-to-background ratio. As a result, to image deep brain regions such as the hippocampus, researchers typically either remove the cortex and then implant a cannula (Robinson et al., 2020) or GRIN lens (Resendez et al., 2016; Sun et al., 2019), both of which, however, may alter the natural brain internal environment. *In vivo* deep optical imaging of mouse brains, such as the hippocampus, is still a major technical challenge in neuroscience research.

Increasing evidence from recent works demonstrate that 3PM is opening up a new way to explore the brain at a deeper level (Horton et al., 2013; Ouzounov et al., 2017; Weisenburger et al., 2019; Wang et al., 2020). 3PM enables hippocampal structural imaging with a depth of more than 1 mm in the mouse brain *in vivo* without invasion. The capability of 3PM in deeper imaging is based on the similar principles that enabled 2PM to become a powerful tool for imaging intact tissues: longer wavelength excitation for reducing the effects of tissue scattering and higher non-linear excitation for suppressing background noise generation. These advantages have sparked an explosion of 3PM research in optical imaging technologies and biological applications (Horton et al., 2013; Ouzounov et al., 2017; Wang T. et al., 2018; Chow et al., 2020). Combined with cutting-edge optical technologies, such as adaptive excitation sources (AES) (Li et al., 2020), adaptive optics (AO) (Rodríguez et al., 2021; Streich et al., 2021; Qin et al., 2022; Sinefeld et al., 2022), and Bessel beam (Chen et al., 2018; Rodríguez et al., 2018), 3PM has made significant advances in imaging performance. In application scenarios, increasing research groups have demonstrated the capabilities of 3PM imaging for probing neural structural and functional dynamics beyond the depth of 2PM, including the applications of 3PM in imaging rodents (Horton et al., 2013; Ouzounov et al., 2017; Wang T. et al., 2018; Liu et al., 2019a; Weisenburger et al., 2019; Klioutchnikov et al., 2020, 2022; Wang et al., 2021; Choe et al., 2022), fishes (Chow et al., 2020; Rodríguez et al., 2021; Akbari et al., 2022a), flies (Tao et al., 2017; Hsu et al., 2019; Aragon et al., 2022), and other kinds of tissues (He et al., 2019; Yildirim et al., 2020; Wang et al., 2021).

In this review, we describe the historical development, advantages, and limitations of 3PM technologies. We then outline the state-of-the-art of 3PM technologies. We also review recent applications. Lastly, we outlook 3PM technology development and applications.

## 2. History of 3PM

**FIGURE 2:**
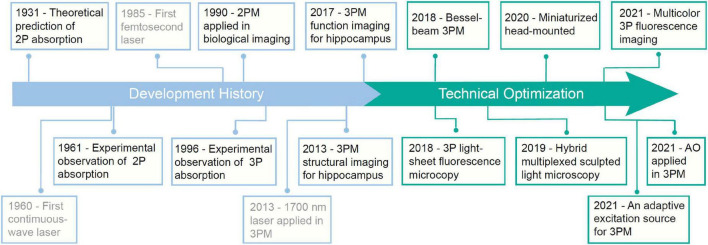
A brief historical timeline of three-photon microscopy (3PM).

The development of the laser propels the development of 3PM (Figure 2). In 1931, Maria Göppert-Mayer first theoretically predicted the process of 2P absorption and emission (Göppert-Mayer, 2009). However, due to the lack of high-intensity monochromatic light sources, the 2P absorption process could not be observed. In 1961, Kaiser and Garrett were able to experimentally verify 2P absorption thanks to the newly invented pulsed ruby laser. However, with a relatively broad pulse width, the pulsed laser cannot provide a sufficiently high peak intensity to excite the fluorescence in biological tissues (Kaiser and Garrett, 1961; Birge, 1983). Fortunately, the advent of femtosecond pulsed lasers in the 1980s removed this stumbling block. With a colliding-pulse, mode-locked dye laser, Denk, Strickler and Webb firstly reported that 2P fluorescence microscope can be applied in biological imaging in Denk et al. (1990). Since then, 2PM has gradually become an indispensable tool in neuroscience research. 3PM was proposed shortly thereafter. In 1996, three groups reported 3PM. Stefan W. Hell and his colleagues reported the evidence of microscopic fluorescence imaging by 3P excitation (Hell, 1996). David L. Wokosin and his colleagues demonstrated 3P imaging of fixed biological samples using an all-solid-state laser (Wokosin et al., 1996). Chris Xu and his colleagues measured the 3P excitation properties of some fluorescent dyes and proteins and demonstrated 3P imaging of rat basophilic leukemia cell stained with DAPI. Because of the high non-linearity, 3PM typically requires high light intensity for excitation. However, due to the lack of high-intensity femtosecond lasers at the most popular 3P spectral window (around 1300 nm and 1700 nm), 3PM has not gotten the deserved attention and extensive application. In 2013, Chris Xu’s group developed a fiber laser with a low repetition rate (1 MHz), high pulse energy (60 nJ), and a longer center wavelength (1,700 nm), and enabled imaging of vascular and hippocampal neuronal structures at a depth of 1.4 mm (Horton et al., 2013). In 2017, the same group used a noncolinear optical parametric amplifier (NOPA) for 3PM imaging of GCaMP6-labelled neurons in the hippocampus of an intact mouse brain (Ouzounov et al., 2017; Wang T. et al., 2018). Today, powerful commercial laser sources are available for 3PMs with high pulse energy (>1,000 nJ), low repetition rates (1–4 MHz), and tunable center wavelengths (1,200–2,500 nm). They greatly facilitate the deeper exploration of the brain.

## 3. Characteristics of 3PM

In this section, we aim to provide a concise overview of the characteristics of 3PM, including the principle and advantages.

### 3.1. Underlying physical principles

**FIGURE 3:**
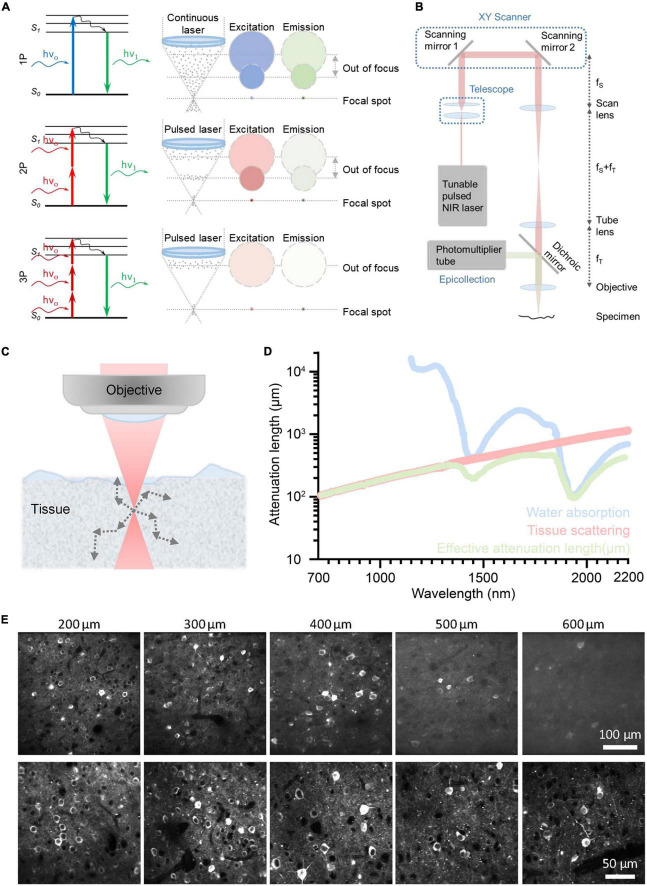
Principle of different optical imaging modalities and a conventional three-photon microscopy (3PM) setup. And the characteristics and performance of two-photon microscope (2PM) and 3PM. **(A)** Left: Jablonski diagrams of one- (top), two- (middle), and three-photon (bottom) fluorescence. Right: A schematic depicting the distribution of laser excitation and fluorescence emission in 1P (top), 2P (middle), and 3P (bottom) excitation scenarios. Gray dots show photons. The repetition rate of the laser source used in 3PM is lower than that of 2PM. In general, the interval of pulses used in 2PM is tens of nanoseconds; The interval of pulses used in 3PM is several microseconds. Circles indicate cross sections of excitation/emission in the x, y plane. Color represents wavelengths and photon intensity. For example, to excite GFP, blue light is used in 1P excitation, long-wavelength light is used in 2P excitation, and longer-wavelength light is used in 3P excitation. **(B)** Schematic of a basic 3PM. Light red shows optical path of excitation light. Green shows optical path. Adapted with permission from Denk et al. (1990). **(C)** Schematic of a focused Gaussian beam in a scattering tissue, with the corresponding axial fluorescence distribution as a function of depth shown in panel **(D)**. **(D)** Wavelength-dependent attenuation length affected by scattering and absorption. Reproduced with permission from Hontani et al. (2022). **(E)** Example images of GCaMP6s with 2P and 3P excitation, focused 200∼600 μm below the pial surface of visual cortex. Adapted with permission from Takasaki et al. (2020).

Figure 3A depicts the energy diagrams of the 1P, 2P, and 3P excited fluorescence processes. The primary distinction between these processes is the number of photons that interact simultaneously with the molecule. In this review, we will only discuss 2P excited fluorescence (Denk et al., 1990) and 3P excited fluorescence (Xu et al., 1996; Bhagwat et al., 1997; McConnell, 2007; Horton et al., 2013) imaging modalities. Additionally, there are two comparable imaging modalities: second-harmonic generation (SHG) (Kompfner and Lemons, 1976; Sheppard et al., 1977; Moreaux et al., 2000; Williams et al., 2005; Rao et al., 2009) and third-harmonic generation (THG) (Barad et al., 1997; Débarre et al., 2006; Farrar et al., 2011; Witte et al., 2011). SHG and THG are also commonly used in multiphoton microscope (MPM). Specifically, SHG and THG are parametric processes (Boyd, 2008) in which there is typically no real energy level involved and the generated signals are exactly at the second- and third-order harmonics of the excitation frequency, respectively.

The majority of fluorophores can be excited by all of these processes, including 1P, 2P, and 3P. Using a longer wavelength is one of the key characteristics of 3P excitation. Compared with 2P excitation, 3P excitation for the same fluorophore uses photons with a lower energy, which corresponds to a longer wavelength, as indicated by the color code of the excitation in Figure 3A. Higher non-linearity is the other crucial characteristic of 3P excitation. Specifically, the intensity of the 3P excited fluorescence is proportional to the cube of the excitation intensity, while the intensity of the 2P excited fluorescence is proportional to the square of the excitation intensity. 3P excitation has two virtual intermediate states, whereas 2P excitation only has one (Xu and Webb, 2002). Typically, the lifetime of the intermediate-state lifetime is on the scale of femtoseconds. Thus, the possibility of simultaneously 3P excitation is much lower than that of simultaneously 2P excitation, that is, the 3P absorption cross section is smaller than 2P absorption cross section. Thus, 3PM typically requires a higher pulse energy (e.g., ∼1 nJ) than 2PM (e.g., ∼0.1 nJ) at the focus (Wang and Xu, 2020). In order to maintain the high pulse energy and safe average power throughout the imaging process, 3PM typically employs a low repetition rate (1–4 MHz versus 80MHz in 2PM) (Horton et al., 2013; Ouzounov et al., 2017; Wang T. et al., 2018).

The configuration of 3PM is similar to that of 2PM, as shown in Figure 3B. The excitation light from the pulsed NIR laser is initially scanned by a pair of scanning mirrors. The scan lens and tube lens are used to conjugate the scanning light onto the objective and adjust the beam size to match the objective back aperture. The objective then focuses excitation light on the specimen. The excited 3P fluorescence is then collected by the objective and dichroic mirror and detected by the photomultiplier tube (PMT). Pre-know scanning knowledge is used by the computer to reform the image. The computer organizes the fluorescence obtained by point-scanning into an image with a known scanning sequence.

### 3.2. Why can 3PM image deeper than 2PM?

#### 3.2.1. A. Long excitation wavelengths increase the penetration depth

As discussed in (Section “3.1. Underlying physical principles”), the 3PM employs long excitation wavelengths, which attenuate less in the brain and result in a large penetration depth. For deep brain imaging, the main obstacle is the scattering and absorption of ballistic photons by the biological tissue (Kou et al., 1993; Theer et al., 2003; Akbari et al., 2022b), as shown in Figure 3C. The scattering of ballistic photons is caused by the heterogeneity of the tissue (Wang M. et al., 2018). Since the intensity of scattering is inversely proportional to the fourth power of the wavelength, the longer wavelengths used for 3PM experience significantly less tissue scattering than the shorter wavelengths typically used for 2PM, as shown in Figure 3D. The absorption in brain tissue is primarily due to water, and the spectral windows around 1,300 nm and 1,700 nm are the local minima of the spectrum of water absorption (Horton et al., 2013; Wang M. et al., 2018), as shown in Figure 3D.

When the light travels in the tissue, the power of the ballistic light can be quantified by P(z) = P_0_.exp(–z/*l*_*e*_), where P(z) is the power at the depth z, P_0_ is the power at the surface, and *l*_*e*_ is the effective attenuation length (Helmchen and Denk, 2005). In brief, the power of ballistic excitation light decreases exponentially with depth in biological tissue. The loss of ballistic photons reduces the generation of fluorescence. To obtain sufficient signal from the focus, the exponential decay of the excitation light needs to be compensated by increasing the optical power at the surface P_0_. However, there is a limit of P_0_ in order to reduce the impact on the physiological environment of the brain (Podgorski and Ranganathan, 2016; Liu et al., 2019b; Yildirim et al., 2019). Therefore, the penetration depth depends on the effective attenuation length *l*_*e*_^14^. It can be further quantified by the formula: *l*_*e*_ = 1/(1/*l*_*s*_ + 1/*l*_*a*_) (Horton et al., 2013), where *l*_*s*_ is the scattering length and *l*_*a*_ is the water absorption length, both of which are wavelength-dependent. The two optimal spectral windows for large effective attenuation length are around 1,300 nm (Ouzounov et al., 2017) and 1,700 nm (Horton et al., 2013) (more details are shown in Figure 3D). In addition, these two spectral windows correspond to the most widely used fluorescent colors, green and red.

#### 3.2.2. B. Non-linear excitation process suppresses the out-of-focus background

The 3PM’s greater non-linearity is crucial for suppressing the out-of-focus background. When the out-of-focus background becomes comparable to the signal generated from the focus, images suffer from low contrast and high background, resulting in a loss of spatial and temporal information. As shown in Figure 3A, the out-of-focus background consists of a bulk background, which is generated in the light cone away from the focus, and a defocus background, which is generated by the side lobes of a distorted point-spread function (Wang and Xu, 2020). For 2PM, the imaging depth is fundamentally limited by the signal-to-background ratio in non-sparsely labeled samples (Theer et al., 2003; Kobat et al., 2011; Cheng X. et al., 2020). For example, its imaging depth is limited to ∼5 effective attenuation lengths for a labeling density of ∼2%, for example, brain vasculature. For 3PM, the background is greatly suppressed by a higher order of non-linear excitation (cubic versus square for 2PM) (Theer and Denk, 2006; Takasaki et al., 2020; Wang et al., 2020), as shown in Figure 3E. For instance, the signal-to-background ratio is greater than 40 even at a depth of ∼5 effective attenuation lengths (∼2,100 μm) (Liu et al., 2019b). Therefore, 3PM at 1,300 and 1,700 nm can be free of background generation for most practical imaging depths.

In conclusion, 3PM allows for a greater imaging depth than 2PM due to its lower scattering at longer excitation wavelengths and higher order non-linear excitation.

## 4. Technical state-of-the-art

**FIGURE 4:**
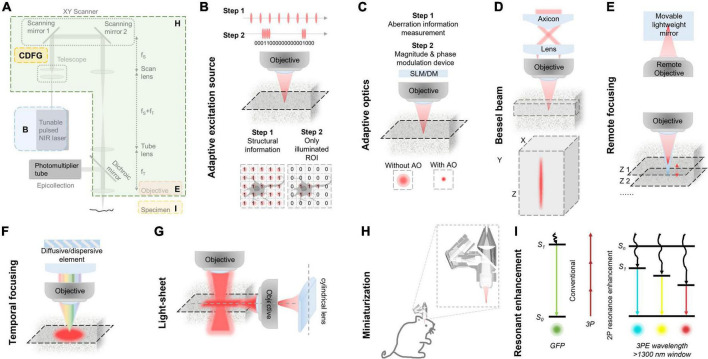
Various improvements in classic three-photon microscopy (3PM) configuration. **(A)** Multiple methods to optimize the performance of 3PM from various perspectives. Different enhancement methods focus on different parts of a 3PM system. **(B)** Simplified schematic of the MPM system with AES. Two steps for the adaptive process. Step 1: Structural images are first captured to acquire the regions of interest (ROI, e.g., the cell body of a neuron) by uniform pulse train. The ROIs information is converted to a digital binary (“0” or “1”) sequence in the time domain and then the ROI information was encoded into the laser pulse pattern. Step 2: The pulse train, which is matched to the sample under study, is amplified to a high pulse energy and launched into the scanning system. With synchronization of scanning and excitation source, the AES only illuminates the ROIs. Gray shadow show ROI morphology. **(C)** A simplified schematic of modules and effect of the AO system. There are two steps for the adaptive optics. Step 1: The focus sensing module measures aberration information. Step 2: The module corrects aberration through magnitude and phase modulation device, such as SLM or DM. With AO correction, the optical resolution in focus was enhanced comparing with the absence of AO correction. **(D)** Top, a simplified schematic representation of an axicon-based Bessel module. Bottom, the Bessel focus exhibits an axial elongation characterized by a needle-like distribution of optical intensity in the XYZ plane. **(E)** Simplified schematic diagram of remote focusing strategy utilized in an imaging system. The strategy typically encompasses a remote objective and a movable lightweight mirror. Without moving the imaging objective and sample, The focal plane can be adjusted by translating the lightweight mirror. A fast actuator can move the mirror at high speed, enabling rapid axial scanning (Z1 to Z2). **(F)** Simplified schematic of temporal focusing strategy used in excitation optical path of an imaging system. This strategy involves incorporating a diffusive or dispersive optical element, such as a grating, into a plane conjugate to the objective focal plane. **(G)** Simplified schematic diagram of light-sheet strategy used in excitation optical path of an imaging system. Generally, light-sheet illumination generated by cylindrical lens on illumination path to achieve widefield imaging. The imaging objective is positioned perpendicular to the illumination objective and focuses on the light sheet to obtain a fluorescence signal. The emitted fluorescence is detected by the different objectives. **(H)** Head-mounted 3PM in a freely behaving rat/mouse. Magnified views of miniaturization configuration of 3PM in dashed box. Adapted with permission from Klioutchnikov et al. (2020). **(I)** Principle of multi-color 3PM. Schematics for simultaneous multi-color 3P excitation of a green (to S1, conventional excitation) and a cyan/yellow/red (to Sn, with 2P resonance enhancement) fluorophore with 1,340-nm excitation. The wiggly arrows indicate non-radiative relaxation. Adapted with permission from Rodríguez and Ji (2021).

The technical community is actively engaged in efforts to enhance the performance of 3PM imaging in multiple dimensions, such as making it faster and wider, in addition to deeper. This section primarily focuses on the advancements and limitations of technology applied to 3PM, as depicted in Table 1. The optimization of 3PM involves various components and recent research has explored various strategies to improve its performance, as illustrated in Figure 4A.

**TABLE 1:** The current technical state of the art in three-photon microscopy (3PM).

Advanced modules	Advantages	Limitations	References
The optimization of 3PM (4.1)	Optimization of Excitation; Fluorescence, Emission, Microscope, Optical power	–	
Adaptive excitation source (4.2)	Less power requirement	System complexity; Improvement coefficient depends on the area of the ROI size relative to the entire FOV	Li et al., 2020
Adaptive optics (4.3)	Better spatial resolution Larger fluorescence signal	System complexity; Requires correction time	Rodríguez et al., 2021; Streich et al., 2021; Qin et al., 2022; Sinefeld et al., 2022
Bessel beam (4.4)	Higher speed for volumetric imaging	Lacks resolution along the axial dimension	Chen et al., 2018; Rodríguez et al., 2018
Remote focusing (4.5)	Faster axial scanning	Used to introduce artificial aberration	Mok et al., 2019; Weisenburger et al., 2019; Klioutchnikov et al., 2022
Temporal focusing (4.6)	Higher frame rate	Tissue penetration capability was restricted	Toda et al., 2017
Light-sheet (4.7)			Escobet-Montalbán et al., 2018
Miniaturization (4.8)	Allows for freely moving	Limited imaging FOV	Klioutchnikov et al., 2020, 2022
Resonant enhancement (4.9)	One wavelength can excite fluorescence of multiple colors at the same time	Only some specific dyes and fluorophores process this property.	Hontani et al., 2021

### 4.1. The optimization of 3PM

#### 4.1.1. Excitation

As discussed in Section 3, the transmittance of excitation light is dependent on its center wavelength (Horton et al., 2013; Cheng et al., 2014). A systematic investigation of *in vivo* 3P imaging at various excitation wavelengths was performed (Wang M. et al., 2018). Theoretical calculations and experiments demonstrated that the optimal 3P excitation spectra are centered around 1,300 and 1,700 nm. The initial femtosecond excitation source was a soliton fiber source with a low-repetition rate and high pulse energy (∼60 nJ) (Wang and Xu, 2011). The center wavelength of the source was approximately 1,700 nm. Polarization multiplexing (He et al., 2017; Tong et al., 2020) or optimized spectral filtering (Wang and Qiu, 2015) were employed to optimize the pulse energy of the soliton fiber source. An alternative fiber source based on self-phase modulation enabled center wavelengths around 1,300 and 1,700 nm (Liu et al., 2016; Chung et al., 2017, 2018; Liu W. et al., 2017) with high pulse energy (>100 nJ). There are also commercially available excitation sources, for example, the non-collinear optical parametric amplifier (NOPA) pumped by a regenerative amplifier (Ouzounov et al., 2017). They offer superior performance for 3PM and have been utilized in many studies (Ouzounov et al., 2017; Wang T. et al., 2018; Weisenburger et al., 2019; Chow et al., 2020; Li et al., 2020; Rodríguez et al., 2021; Streich et al., 2021; Choe et al., 2022).

#### 4.1.2. Fluorescence

The efficiency of 3P absorption is dependent on the absorption cross section of the fluorophore. The 3P absorption cross section spectra of many fluorescent proteins and dyes were measured (Deng et al., 2019; Liu et al., 2020, 2018). These studies provide an excellent guide for selecting fluorescence tools and the corresponding center wavelength of the excitation laser. To further increase the absorption cross section, new dyes (Ren et al., 2019) and quantum dots (Liu et al., 2019b; Hontani et al., 2021) were also used for 3PM. The 3P absorption cross-section of quantum dots is 4–5 orders of magnitude larger than that of conventional fluorescent dyes, allowing for a brain imaging depth of up to 2,100 μm.

#### 4.1.3. Emission

It has been known that the fluorescence emission at longer wavelengths scatters less in the mouse brain. Nonetheless, a study revealed that the emission wavelength does not significantly influence the fluorescence collection efficiency (Wang et al., 2019). When using wide-field geometry for fluorescence collection, both ballistic photons and scattered photons out of the brain would be collected. Therefore, a wide-field geometry fluorescence collection system can be used to diminish the effect of scattering length in emission.

#### 4.1.4. Microscope

As the center wavelength of excitation for 3PM is longer than that of conventional 2PM, the optics of the microscope need to be coated at the 3P excitation window. The transmittance of objective lens (Wen et al., 2018) and immersion medium (Wang Y. et al., 2016; Liu H. et al., 2017; Tong et al., 2019; Zhuang et al., 2019) was examined and optimized for 3PM. The selection of PMT is also a factor in achieving higher response to collected photons (Wang K. et al., 2016). In general, GaAsP PMT works better for fluorescence at shorter wavelengths, including majority of the conventional fluorescent colors, i.e., blue, green, yellow, and red. GaAsP PMT is superior for fluorescence at longer wavelengths, for example, quantum dots Qtracker 655.

#### 4.1.5. Optical power threshold for biosafety

To minimize the impact on the brain, it is essential to confine the laser power to a safe range. A study revealed that there is no evidence of damage when the power is under 100 mW at 1,300 nm for imaging at a depth of 1 to 1.2 mm by 3PM (Wang et al., 2020). The safety threshold of 3PM is lower than that of 2PM at 920 nm, which is ∼ 250 mW (Podgorski and Ranganathan, 2016). The primary reason is that 1,300 nm light has a higher absorption efficiency in the brain. Aside from brain heating, the pulse energy of the excitation must be kept low to prevent tissue non-linear damage, that is optical breakdown caused by the non-linear effects of laser, in the focus. The non-linear process of optical breakdown begins with the generation of free electrons through a combination of multiphoton ionization and band-gap (Zener) tunneling, resulting in a subsequent breakdown event in the sample (Joglekar et al., 2004; Vogel et al., 2005). A study found that the safety range of the pulse energy for 3PM at 1,300 nm is 0–5 nJ (Yildirim et al., 2019). The optimization of average power and pulse energy while maintaining safety limits from brain heating and non-linear effects was studied (Wang and Xu, 2020).

### 4.2. Adaptive excitation source (AES): Lower power requirement

Adaptive excitation source (AES) is a laser source that reduces the power requirement for 2P or 3P imaging of brain activity in awake mice for improved high-speed neuroimaging (Li et al., 2020). As shown in Figure 4B, in the MPM system with AES, structural images are captured to acquire the regions of interest (ROI, e.g., the cell body of a neuron) by uniform pulse train in the first step. The ROIs information is converted to a digital binary sequence in the time domain and then fed to an arbitrary waveform generator. The arbitrary waveform generator drives a fiber-integrated electro-optic modulator (EOM) and encodes the ROI information into the laser pulse pattern. The pulse train of the second step, which is matched to the sample under study, is amplified to a high pulse energy and launched into the scanning system. With synchronization of scanning and excitation source, the AES is able to allocate all the permissible laser power to the ROIs by removing the “unwanted” pulses outside the ROIs. Consequently, it is possible to increase imaging speed and the number of neurons at low light exposure.

The AES has been successfully implemented in the 3PM system (Li et al., 2020). Researchers used the 3PM with AES to perform *in vivo* imaging on a transgenic mouse that was densely labeled with the GCaMP6 indicator. Using a laser power of 35 mW, the system imaged jRGECO1a-labeled neurons at 750 μm beneath the dura in an awake mouse with a field of view (FOV) of 620 μm× 620 μm (512 × 512 pixels/frame), and an imaging speed of 30 Hz. Traditional laser sources would require approximately 1,000 mW to achieve the same results, which is way above the threshold for brain damage in mice. The imaging strategy achieves a combination of a large depth, a large FOV, and a high speed in imaging performance. Additionally, AES is compatible with existing multiphoton microscopes, without requiring any extra hardware modifications.

### 4.3. Adaptive optics: Better spatial resolution

Adaptive optics (AO) is a technique for measuring and correcting the distortion of excitation wavefront in scattering tissues in order to recover optical spatial resolution and boost fluorescence signal intensity and contrast. AO could reduce the aberration caused by heterogeneous brain tissue. As shown in Figure 4C, the first step of AO correction is to determine the excitation wavefront using either direct (Wang et al., 2014, 2015; Liu et al., 2019c) or indirect (Débarre et al., 2009; Ji et al., 2010; Tang et al., 2012; Wang C. et al., 2014) approaches; and the second step is to correct the specimen-induced aberrations using a phase modulation device, such as spatial light modulator (SLM) or deformable mirror (DM).

Recent studies have demonstrated AO correction in the 3PM region of the mouse brain. Robert Prevedel’s group developed a continuous membrane deformable mirror (DM) and a modal-based AO optimization scheme with automatic shift correction (Streich et al., 2021). The system employs a sequential Zernike polynomial correction method, which typically requires 20–30 s for measurement. The modal-based, sensorless AO approach is robust to low signal-to-noise ratios, which are commonly encountered in deep scattering tissues such as the mouse brain and allows AO correction over large axial fields of view. In addition, the group implemented a prospective image-gated acquisition scheme based on a field-programmable gate array (FPGA), which enables active synchronization of the scanners to the cardiac cycle in real time so that active scanning is paused during peaks of ECG recording. The design reduces intraframe motion artifacts and enables the acquisition of a more stable temporal image. The authors observed a 4 × improvement in effective axial resolution and about 8 × enhancements in fluorescence signals. The axial resolution of the system is 3.1 and 7.9 μm in the cortex (depth at 653 μm) and hippocampus (depth at 1,054 μm), respectively. The ECG-gated 3P-AO scope can reliably resolve individual synapses down to 900 μm in the cortex and fine dendritic processes as deep as 1.4 mm in the hippocampus. The authors also showed applications to deep-layer calcium imaging of astrocytes, including fibrous astrocytes that reside in the highly scattering corpus callosum. Recently, Chris Xu’s group (Sinefeld et al., 2022) also used similar methods to correct the wavefront aberration in 3PM with a comparable imaging performance.

Na Ji’s group developed a compact AO module that implemented the frequency-multiplexed aberration measurement and the subsequent correction using only a deformable mirror (DM) (Rodríguez et al., 2021). The module permits high power throughput and polarization- and wavelength-independent operation. Under a broad range of excitation wavelengths, the compact AO module can be added to a 2P or 3P fluorescence microscope. Moreover, the compact AO module is based on a zonal aberration measurement method, similar to the pupil segmentation method (Ji et al., 2010), which typically requires 20–30 s for measurement. Using 1,300 nm excitation, the AO module improved the 3P fluorescence signal of YFP-labeled neurons by 4 × on the cell body and 2–7 × on dendritic structures, at 700 μm below dura. In addition, at a depth of 310 μm below the dura in the mouse spinal cord, the authors observed that aberration correction significantly enhanced the signal of a neuronal cell body (by 5.9 ×) and increased the peak calcium-dependent fluorescence change (ΔF/F_0_, by 2.1 ×).

The preceding three implantations are based on indirect AO approaches. Recently, Jianan Y. Qu’s group developed an AO technique based on analog lock-in phase detection for focus sensing and shaping (ALPHA-FSS). The approach measures the aberrated electric field point spread function (E-field PSF) directly. They introduced a high-frequency modulation and phase-sensitive detection scheme to achieve accurate measurement of the E-field PSF in a fast and photon-efficient manner, which allows subsequent aberration correction of a large number of modes by using a high-pixel-count wavefront corrector. In addition, they integrated a remote focusing approach with the conjugate AO configuration to enable effective single correction over a large volume for imaging through a turbid layer. The effective FOV after a single correction of conjugate AO is extended to 150 and 400 μm in the lateral and axial directions, respectively. The system effectively recovered high imaging resolution to 4.8 μm in the axial direction and improved the fluorescence intensity more than 200-fold. The system enables *in vivo* imaging of fine neuronal structures in the mouse cortex through the intact skull at a depth of 750 μm below the pia, allowing for near-non-invasive high-resolution microscopy in the cortex. In addition, the authors also achieved *in vivo* high-resolution functional imaging of the deep cortex and subcortical hippocampus as deep as 1.1 mm below the skull window.

### 4.4. 3PM with Bessel beam: Higher axial throughput

Bessel beam scanning is a rapid volumetric imaging method for sparsely labeled samples with an axially elongated Bessel focus (Thériault et al., 2013; Lu et al., 2017, 2020; Fan et al., 2020; Chen et al., 2021). As shown in Figure 4D, the elongated Bessel focus has an axially elongated, needle-like optical intensity distribution. Thus, the same lateral resolution is maintained throughout the depth of field. Typically, the Bessel focus is achieved by illuminating the back focal plane of the microscope objective with an annular pattern. When the Bessel focus is scanned laterally in two dimensions, a 2D projection of the 3D volume image is captured, where the 3D volume is determined by the 2D scanning area and the axial length of the Bessel focus. Therefore, Bessel beam scanning has a 3D volume rate that is comparable to the 2D frame rate of conventional point-scanning techniques. It has been demonstrated that this method works well with sparsely labeled, high-contrast structures (Botcherby et al., 2006; Thériault et al., 2014; Yang et al., 2016). However, this technique lacks resolution along the axial dimension, making it not suitable for imaging of densely labeled samples.

Bessel focus scanning has been successfully applied in 3PM for rapid volumetric imaging (Chen et al., 2018; Rodríguez et al., 2018). Na Ji’s group (Rodríguez et al., 2018) obtained *in vivo* structural images of a 30-μm thick volume from the Gad2-IRES-Cre: Ai14 mouse, at ∼290 μm below the dura. Under 1,700 nm excitation, these images were obtained with a Bessel focus of NA 0.6, an axial FWHM of 27 μm, and power of 73 mW. Aimin Wang’s group (Chen et al., 2018) built a 3PM with Bessel beam scanning, and demonstrated imaging of a volume of 300 × 300 × 65 μm^3^, with a deep penetration (620 μm below the dura) and high speed (1 Hz, 512 × 512 pixels/frame). Theoretically and experimentally, they demonstrated the higher signal-to-background ratio (SBR) of the Bessel-beam 3PM compared to the 2P version.

### 4.5. 3PM with remote focusing: Faster axial scanning

Remote focusing is an optical refocusing technique that is implemented remotely from the specimen (Anselmi et al., 2011; Thériault et al., 2014; Cheng Z. et al., 2020; Jiang et al., 2020; Durst et al., 2021). As shown in Figure 4E, this module typically uses a remote objective and a movable lightweight mirror to refocus the imaging plane within the specimen (Schuetzenberger and Borst, 2020). When the mirror moves perpendicularly to the light, the imaging plane will shift relative to the nominal focal plane. The scheme does not require moving a relatively heavy imaging objective and enables faster axial focus movement.

Remote focusing has been implemented in multiple 3PM systems. It was demonstrated that a dual-plane 3PM can simultaneously image two planes in the mouse brain, 600 and 650 μm below the dura (Takasaki et al., 2019). The remote focusing module controls the refocusing of one plane and provides a tuning range of ± 50 μm. The remote focusing was also applied in a conjugated-AO 3PM, enabling the effective improvement of imaging resolution over a large imaging depth of 400 μm, with a single corrective wavefront (Qin et al., 2022). The remote focusing strategy has also been successfully implemented in a dual-plane 2P and 3P imaging system, where 2PM and 3PM image the shallow and deep planes, respectively (Weisenburger et al., 2019; Takasaki et al., 2020). In one work, a remote focusing module was developed to independently control the refocusing of two planes by a movable dichroic mirror and a mirror (Mok et al., 2019). The beam at 920 nm for 2PM was reflected by the dichroic mirror and thus, the refocusing can be realized by shifting the dichroic mirror. And the beam at 1,320 nm for 3PM passes through the dichroic mirror, and the refocusing was accomplished by shifting the movable lightweight mirror. In the mouse brain imaging experiment, 2PM and 3PM captured the calcium signals of neurons from cortical layer 2/3 (330 μm depth) and layer 5 (630 μm depth), respectively. In another work, only the beam at 910 nm for 2PM is remotely tunable by using an electrically tunable lens (Takasaki et al., 2020). Recently, remote focusing was implanted into a miniature 3PM so that the imaging plane could be adjusted without disturbing the animals behavior (Klioutchnikov et al., 2022).

### 4.6. 3PM with temporal focusing (TF): Higher frame rate

Temporal focusing provides a means of axially modulating the photon density to achieve axial confinement by multiphoton excitation (Oron et al., 2005; Durst et al., 2006; Papagiakoumou et al., 2010, 2020; Pégard et al., 2017; Mardinly et al., 2018). As shown in Figure 4F, temporal focusing uses a diffusive or dispersive optical element, such as a grating, positioned in a plane conjugate to the objective focal plane. In this way, the beam at out-of-focus planes is dispersed, whereas the beam at the objective focal plane is not. Consequently, the rapid reduction of multiphoton absorption away from the focal plane results in optical sectioning. In this modality, fluorescence from the excited plane is collected by epi-detection and a camera. The method helps MPMs, which mainly use point scanning mode, overcome the limited temporal resolution of excitation, and enables a high imaging speed. Meanwhile, the efficiency of the widefield excitation and strong scattering of the emission fluorescence restrict the imaging depth.

Recently, temporal focusing has been used in 3PM. In one work (Toda et al., 2017), the authors demonstrated that the optical sectioning capability of 3P temporal-focusing microscopy was improved from 2.1 to 1.6 μm with 92-fs 9.0-μJ 1,060-nm pulses at a repetition rate of 200 kHz. In addition, dual-color imaging of a fixed mouse brain sample stained with DAPI or SYTO83 at a depth of 30 μm was performed. In another study (Rowlands et al., 2017), the widefield 3P temporal-focusing microscopy imaged quantum dots in fixed brain slices with a penetration depth of 800 μm. 3P excitation at 1,300 nm successfully induced action potentials in cultured neurons expressing the optogenetic protein CoChR. The used power is 64 ± 11 mW.

### 4.7. Light sheet 3PM: Higher frame rate

Light-sheet technology is an illuminated technique that uses a thin sheet of light to excite only fluorophores within the focal volume and a camera to capture the image focal plane. The simplest way to produce a light sheet is to introduce a cylindrical lens into the illumination path, as shown in Figure 4G. The imaging objective is positioned perpendicular to the illumination objective and focuses on the light sheet to obtain a fluorescence signal. The emitted fluorescence is detected by the different objectives which lead to the camera. This technique has multiple merits, such as high speed due to widefield imaging, low background and phototoxicity due to light-sheeting illumination.

In 2018, the light-sheet fluorescence microscope using 3P excitation was first reported (Escobet-Montalbán et al., 2018). The authors used a femtosecond pulsed laser at 1,000 nm wavelength for the imaging of cellular spheroids labeled with PUREBLU Hoechst 33,342 nuclear staining dye. 3P excited light-sheet microscope has a higher contrast-to-noise ratio than that of 2P for deep imaging. The contrast-to-noise ratio in 2P mode drops by approximately 71% at a depth of nearly 450 μm while in 3P mode it only decreases by 15%. However, for 3PM, the imaging depth is limited by the efficiency of the widefield excitation and the strong scattering of the emission fluorescence.

### 4.8. Miniaturized 3PM: Head-mounted and freely behaving

The head-mounted multiphoton microscope is a miniaturized imaging tool that is suitable for monitoring the structural and functional dynamics of the brain in freely moving animals, as shown in Figure 4H (Piyawattanametha et al., 2009; Ghosh et al., 2011; Zong et al., 2017, 2021). Researchers typically use optical fibers for fluorescence excitation and collection and use microelectromechanical system (MEMS) scanning mirrors for laser scanning.

Klioutchnikov and his colleagues developed a miniaturized 3PM for freely moving rats (Klioutchnikov et al., 2020). They designed a hollow-core photonic bandgap crystal fiber (HC-PBGF) to maintain the intensity and polarization state of the excitation light when the animal moves or the fiber bends. The miniaturized 3PM weighs 5.0 g, making it suitable for rats and larger animals, but too heavy for mice. The system can image neurons and dendrites to a maximum depth of 1,120 μm below the cortical surface. It also enables recording the calcium activity of the neuronal populations at 950 μm below the cortical surface when the animal is freely moving, with a FOV of 140 × 140 μm (120 × 120 pixels/frame) and an imaging speed of 28 Hz. Recently, the same group built 2-g miniature 3PM that is bearable for freely behaved mice (Klioutchnikov et al., 2022). With the 3PM, neurons and vascular structures were observed in mice from layer 1 to the corpus callosum. In addition, the entire cortical mantel could be accessed *via* the Z-drive remote control. This optical configuration has the FOV of 300 μm× 300 μm (273 × 280 pixels/frame) (limited by the scanner specifications), and an imaging speed of 10.6 Hz., The system offers lateral resolutions of 1.0–1.2 μm and axial resolutions of 9–19 μm in the full focus range.

### 4.9. 3PM with resonant enhancement: Multi-color parallelly imaging

Resonance enhancement is a technique for blue-shifted excitation to a higher-energy electronic excited state, as opposed to conventional excitation to the lowest-energy excited state (Hales et al., 2004; Drobizhev et al., 2007, 2011; Sadegh et al., 2019), as shown in Figure 4I. Due to the enhancement effect, the method could enhance the fluorescent molecular excitation cross section of some common red fluorescent molecules.

Chris Xu’ group recently reported the resonance enhancement of 3P excitation and simultaneous imaging of green and red fluorescent molecules using a single wavelength at the 1,300-nm spectral window (Hontani et al., 2021). They found that the peak of the 3P excitation spectrum of some fluorescent molecules can be blue-shifted relative to the peak of the one-photon excitation spectrum (with the 1P excitation wavelength scaled by 3 ×) by hundreds of nanometers. They also demonstrated that the blue-shifted 3PE cross sections in the 1,300-nm window are more than 10 times larger than those in the 1,700-nm window for some red fluorophores, which greatly enhances the signal strength in 3PM. By using a single excitation wavelength at 1,340 nm, they demonstrated the multicolor imaging of different cell populations that were simultaneously captured up to 1,100 μm deep in the mouse brain, including CFP-labeled oligodendrocytes, EGFP-labeled microglia, YFP-tagged neurons, and DsRed-Max-tagged astrocytes (Hontani et al., 2021). In addition, they simultaneously recorded the calcium activity of GCaMP6s-labeled neurons and the structure of sulforhodamine101-labeled astrocytes at 762 μm below the dura. This method enables multi-color 3PM with a single excitation wavelength, which could be used to observe molecular and cellular interactions in the brain and other organs over a large depth *in vivo* in real time.

## 5. Application of 3PM

**FIGURE 5:**
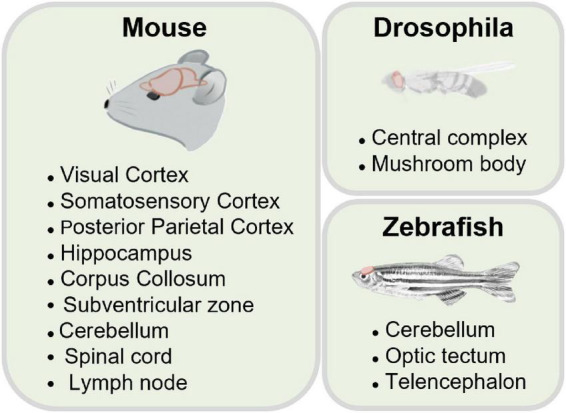
Application in various samples.

Recently, the neuroscience community has tried 3PM out to perform minimally invasive three-dimensional imaging *in vivo* with cellular resolution in a variety of samples. In the following section, we will focus on the successful applications of 3PM in living biological samples (Figure 5).

### 5.1. Rodents

#### 5.1.1. Hippocampus

**FIGURE 6:**
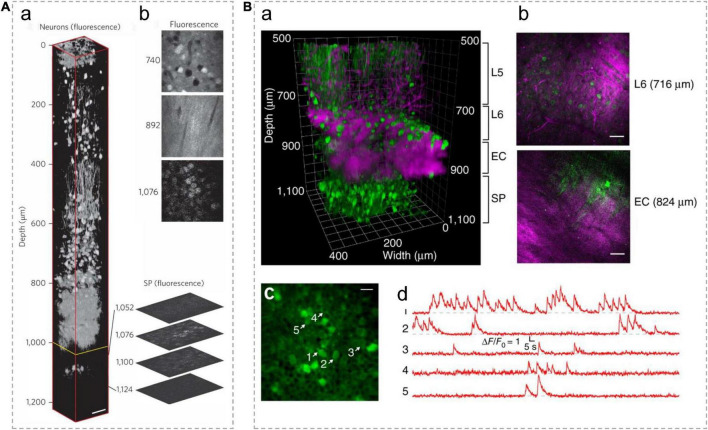
*In vivo* 3P imaging in the mouse hippocampus. **(A)** (a) A 3D reconstruction of mouse brain images captured using 3PM, with pyramidal neurons labeled by RFP. The frames deeper than 992 μm (yellow line) were normalized to the frame at 1,076 μm and all other frames were individually normalized. Expanded optical sections showcase representative fluorescence images of the SP layer. (b) Normalized fluorescence images at various depths. Scale bar, 50 μm. Adapted with permission from Horton et al. (2013). **(B)** (a) A 3D reconstruction of 3PM images of neurons labeled with GCaMP6s in the mouse cortex and the hippocampus. (b) Selected frames at different depths in a show blood vessels and myelinated axons with THG signal. Scale bars, 50 μm. (c) Activity recording locates in the SP layer of the hippocampus (at 984 μm beneath the dura) with a FOV of 200 μm× 200 μm. Scale bar, 20 μm. Green indicates fluorescence signal; Magenta indicates THG signal. L5/6, layer 5/6 in cortex; EC, external capsule; SP, stratum pyramidale. (d) Spontaneous calcium signal recorded from the labeled neurons indicated in c. Adapted with permission from Ouzounov et al. (2017).

The hippocampus is one of the key brain areas for learning and memory. Previous studies using 2PM typically remove above cortical tissue (Dombeck et al., 2010; Robinson et al., 2020) or insert optical elements to access hippocampus (Levene et al., 2004; Low et al., 2014; Keinath et al., 2020). These procedures usually cause irreversible damage to the brain. Several exciting studies have demonstrated that 3PM can image the hippocampus in an intact brain. In 2013, for the first time, 3PM was used to successfully capture structural imaging of the hippocampus within an intact mouse brain (Horton et al., 2013), as shown in Figure 6A. Under 1,700 nm excitation wavelength, the authors obtained high-resolution and high-contrast images of the blood vessels up to 1,300 μm deep through a cranial window. The vasculature was labeled with dextran-coupled Texas Red dye. The used power was 22 mW which is far lower than the acceptable power limit of mouse brain tissue. They also imaged the red fluorescent protein (RFP)-labeled pyramidal neurons in the stratum pyramidale (SP), a dense layer of pyramidal neurons within the CA1 area of the hippocampus (1,060–1,120 μm below the surface of the brain). The 3PM system acquired images with a FOV of 123 μm× 123 μm (512 × 512 pixels/frame). The upper bounds of the lateral resolution and axial resolution of the system at the depth of around 1 mm are respectively ∼0.9 and ∼4.4 μm.

Ouzounov and his colleagues first demonstrated that 3PM enables functional imaging of neurons in the hippocampus within intact mouse brains (Ouzounov et al., 2017), as shown in Figure 6B. They expressed the classical calcium indicator, GCaMP6s, by adeno-associated virus (AAV) into neurons in the SP layer (∼1 mm beneath the dura) of the mouse hippocampus. Under 1,300 nm excitation, spontaneous activities of these neurons were recorded with an average power of ∼50 mW under the objective lens. The fluorescence images were achieved with a FOV of 200 μm× 200 μm (256 × 256 pixels/frame) at a 8.49-Hz frame rate, which are typical parameters used for capturing neuronal activities of GCaMP6s-labeled neurons *in vivo*. This work demonstrates that the imaging performance of 3PM at deep brain regions is comparable to that of 2PM at shallower imaging depths of the mouse brain. This method makes it possible to record the activity of neurons non-invasively with high spatial and temporal resolution deep in the brain.

The system of 3PM can be modified to acquire high throughput and fast speed imaging in deep brain areas. By slightly lowering the spatial resolution and increasing the laser power in 3PM, a recent published work (Weisenburger et al., 2019) simultaneously imaged neuronal activities of 100–150 neurons from a FOV of 340 μm× 340 μm (100 × 100 pixels/frame, the lateral pixel size was ∼3.4 μm) at a maximum of 78 Hz per single-plane from depths up to 1.22 mm. Under 1,300 nm excitation wavelength, the highest average power used to image the deepest plane was ∼190 mW. Furthermore, the optimized 3PM can perform volumetric recordings. The calcium activities of layer 6b neurons in the posterior parietal cortex and hippocampus CA1 were recorded at 3.9 Hz within a ∼340 × 340 × 250 μm^3^ volume located between 750 and 1,000 μm below the brain surface. The lateral and axial resolution of the system are ∼1.5 and ∼9.4 μm, respectively.

3PM with the AO correction module enables imaging of hippocampal structures with subcellular resolution (Rodríguez et al., 2021; Streich et al., 2021; Qin et al., 2022; Sinefeld et al., 2022). In a previous work, when applying the AO correction module, the lateral and axial resolutions of the 3PM system are, respectively improved to ∼0.6 and ∼2.3 μm, which allows successful imaging of YFP-labeled dendritic spines at ∼760 μm below the dura in the transgenic mouse cortex (Rodríguez et al., 2021). The system is also compatible with dual-channel imaging. By switching the excitation wavelength to 1,700 nm, tdTomato-labeled neuronal cell bodies can be imaged at 952∼1,020 μm below the dura. The recorded neurons in the hippocampus were labeled with YFP/tdTomato in transgenic mice or infected with AAV virus carrying a Cre-recombinase-dependent tdTomato element in wild type mice. Under 1,300 nm excitation, the used average power is 10∼40 mW. The system acquired images with a FOV of 200 μm× 200 μm. In another work, 3PM with AO correction was able to observe hippocampus CA1 neurons and dendrites processes (Streich et al., 2021) at even deeper location (1.45 mm below the dura), which reaches the edge of the stratum lacunosum moleculare in the dorsal hippocampus. The axial resolution of the 3PM system is 4–6 μm beyond depths of 1 mm. The imaged neurons were labeled by EGFP in transgenic mice. The system scans a FOV of 126 μm× 126 μm (256 × 256 pixels/frame). AO also facilities skull-intact imaging to minimize brain damage. 3PM with AO and remote focusing modules can clearly image apical dendritic spines of hippocampal CA1 neurons at depths up to 1,140 μm below pia through the intact skull (Qin et al., 2022). These experiments were performed in Thy1-GFP mice. The used post-objective power is 64 mW in the deepest imaging plane, under 1,300 nm excitation. The system scans a FOV of 120 μm× 120 μm (128 × 128 pixels/frame).

#### 5.1.2. Glia cells

**FIGURE 7:**
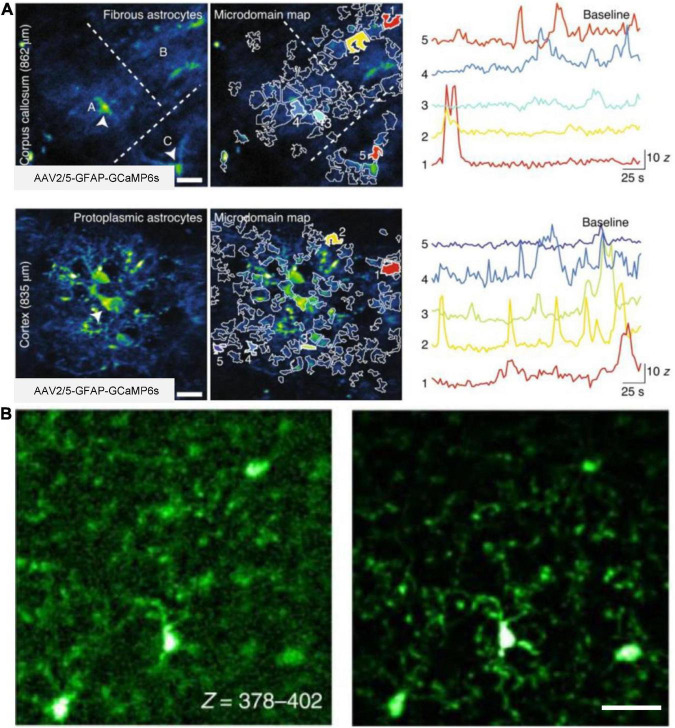
*In vivo* 3P imaging of astrocytes and microglia in the mouse brain. **(A)** Imaging of Ca^2+^ activity of fibrous and protoplasmic astrocytes in the white and gray matter. Top, median intensity time-series projection image of three fibrous astrocytes (A–C) in the corpus callosum (862 μm beneath dura). Arrowheads show astrocyte soma (left). Map of selected active microdomains (middle). Intensity versus time traces for five microdomains, which corresponding to colors in middle panel (right), displaying characteristics of Ca^2+^ transients. Bottom, similar to top, but for astrocytic Ca^2+^ imaging in the layer 6 of the visual cortex (835 μm beneath dura). Adapted with permission from Streich et al. (2021). **(B)** Maximum intensity projection images of GFP-labeled microglia in a Cx3Cr1-GFP transgenic mouse. Images captured at a depth of 378–402 μm, without AO (left) and with full AO (right) correction through the intact skull. Scale bar, 10 μm. Adapted with permission from Qin et al. (2022).

3PM are also used to study glial cells in several recent studies. One study used 3PM excited at 1,700 nm to visualize astrocytes labeled with sulforhodamine101 at a depth of 910 μm below the mouse brain surface. The image depth of 3PM is 30% deeper than previous results of 2PM (Liu et al., 2019a). Furthermore, the other work demonstrated the capability of AO-3PM to detect Ca^2+^ transients with high SBR in individual microdomains of GCaMP6f-labeled astrocytes located in the corpus callosum in mouse brain (up to 862 μm) (Streich et al., 2021), as shown in Figure 7A. Using 3PM at the 1,700-nm excitation window, another work visualized the microglia tagged with Dylight 649 at 1,124 μm below the brain surface (Cheng H. et al., 2020). Images were acquired at an acquisition rate of 25 Hz with a FOV of 40 μm× 40 μm (64 × 64 pixels/frame). Additionally, in a recent work (Qin et al., 2022), 3PM with AO enabled *in vivo* imaging of GFP-labeled microglia in adult Cx3Cr1-GFP mice, as shown in Figure 7B. Moreover, a recent work showed multicolor 3PM imaging using single-wavelength excitation (Hontani et al., 2021). Upon 1,340 nm excitation, 3PM can simultaneously acquire fluorescence images of GCaMP6s-labeled neurons, Texas Red-labeled blood vessels (or sulforhodamine101-labeled astrocytes), and THG up to 1,200 μm deep in the mouse brain. The GCaMP6s signals were captured with a FOV of 270 μm× 270 μm (256 × 256 pixels/frame) at a frame rate of 8.3 Hz. Additionally, the work obtained multicolor brain images using a PrismPlus mouse expressing cyan fluorescent protein (CFP; Cerulean) in oligodendrocytes, enhanced green fluorescent protein (EGFP) in microglia, yellow fluorescent protein (YFP) in neurons, and DsRed-Max in astrocytes with a single excitation wavelength at 1,340 nm. In these experiments, the maximum average power under the objective was 70 mW. Therefore, 3PM is highly beneficial in the morphometric analysis and functional surveys of calcium dynamics in glial cells.

#### 5.1.3. Intact skull imaging

**FIGURE 8:**
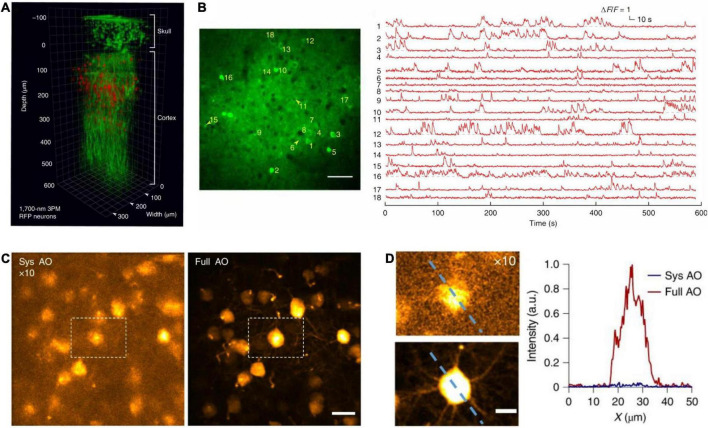
*In vivo* cortical 3P imaging through the intact skull. **(A)** 3D reconstruction of CaMKII-positive neurons (∼140 μm below the cortical surface) labeled with GCaMP6s in a transgenic mouse. Images were captured by 1,320 nm 3PM through an intact skull of ∼100 μm thickness (red, fluorescence; green, THG). **(B)** Imaging site for through-skull activity recording in an awake, GCaMP6s-labeled transgenic mouse. The recording site was ∼275 μm beneath the dura, and the FOV was 320 μm× 320 μm (256 × 256 pixels per frame). Scale bar, 50 μm. **(A,B)** Were adapted with permission from Wang T. et al. (2018). **(C)** Maximum intensity projection of stack images (at the depth of 657–747 μm) of pyramidal neurons without (left) and with full AO correction (right). Scale bar, 20 μm. **(D)** Zoom in neurons in white dashed box in panel **(C)** (left). Scale bar, 10 μm. Signal intensity along the dashed line on left panel (right). a.u., arbitrary units. **(C,D)** Were adapted with permission from Ref. Qin et al. (2022).

Deep brain imaging typically requires skull thinning or cranial craniotomy. Recent research has shown that 3PM allows imaging mouse brains with intact skulls. The absence of skull thinning, or cranial craniotomies minimizes physiological intrusion. In 2018, 3PM achieved fluorescein-labelled vascular imaging at depths greater than 500 μm with an intact skull (Wang T. et al., 2018), as shown in Figures 8A, B. Moreover, 3PM can also record calcium activity of cortical layer 4 CaMKII-positive neurons located at a depth of up to 465 μm below the cortical surface with an intact skull (Wang T. et al., 2018). The neurons are labeled by GCaMP6s in transgenic mice. Under 1,300 nm excitation, the average power used for imaging was 44 mW. The FOV was 320 μm× 320 μm (256 × 256 pixels/frame). The images were acquired at a frame rate of 8.49 Hz. The lateral and axial resolution were 0.96 and 4.6 μm, respectively.

Besides, in a recent work (Qin et al., 2022), 3PM with AO enables *in vivo* imaging of fine neuronal structures in the Thy1-YFP transgenic mouse cortex through the 100-μm-thickness intact skull up to a depth of 750 μm below the pia, as shown in Figures 8C, D. The researchers also obtained *in vivo* images of GFP-labeled microglia at 400 μm depth in adult Cx3Cr1-GFP mice. Moreover, the researchers performed *in vivo* calcium imaging of GCaMP6s-labeled CCK-neurons at 400 μm depth in mouse somatosensory cortex through the intact skull. The images were acquired at 4.43 frames/s. In these experiments, under 1,300 nm excitation, the average power used for imaging was less than 100 mW. For both functional and structural imaging, the FOV was 50 μm× 50 μm (256 × 256 pixels/frame). And the axial resolution was 3.9∼7.2 μm and deteriorated while increasing depth.

#### 5.1.4. Spinal cord

**FIGURE 9:**
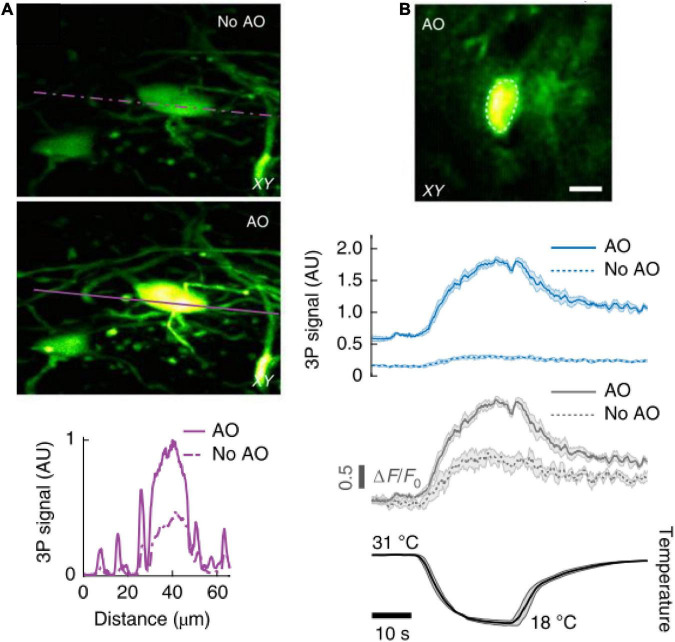
*In vivo* 3P imaging in the mouse spinal cord. **(A)** Maximum intensity projection of spinal cord neurons (Thy1-GFP transgenic mouse) located 208–228 μm below dura, under 1,300 nm excitation, without (top) and with (bottom) AO correction. Signal intensity the purple lines. **(B)** AO corrected image of a jGCaMP7s-expressing neuron (310 μm below dura) of the dorsal horn in the mouse spinal cord, under 1,300 nm excitation (top). 3P fluorescence signal (middle) and calcium transients (ΔF/F0) (bottom), in response to cooling stimuli, without and with AO correction. Average traces from 4 trials; shaded area, s.e.m. Adapted with permission from Rodríguez et al. (2021).

As a part of the central nervous system, the spinal cord is relatively hard to image due to the high neuronal density and the strong optical scattering caused by the superficial distribution of axon tracts. In a recent work (Rodríguez et al., 2021), by incorporating an AO module with 3PM, the authors performed *in vivo* imaging of GFP-labeled neuronal structures at depths exceeding 400 μm below the dura in adult transgenic mice through a dorsal laminectomy. Under 1,300 nm excitation light, the used post-objective power was 89 mW. Moreover, the advanced system also reliably recorded calcium transients in jGCaMP7s-expressing neurons of the dorsal horn (at depths beyond 310 μm) in the mouse spinal cord in response to cooling stimuli applied to the skin of the hindlimb, as shown in Figure 9. In the experiment, the neurons were labeled by fluorescence through viral delivery methods. The post-objective power was merely 4.2 mW.

#### 5.1.5. Lymph node

**FIGURE 10:**
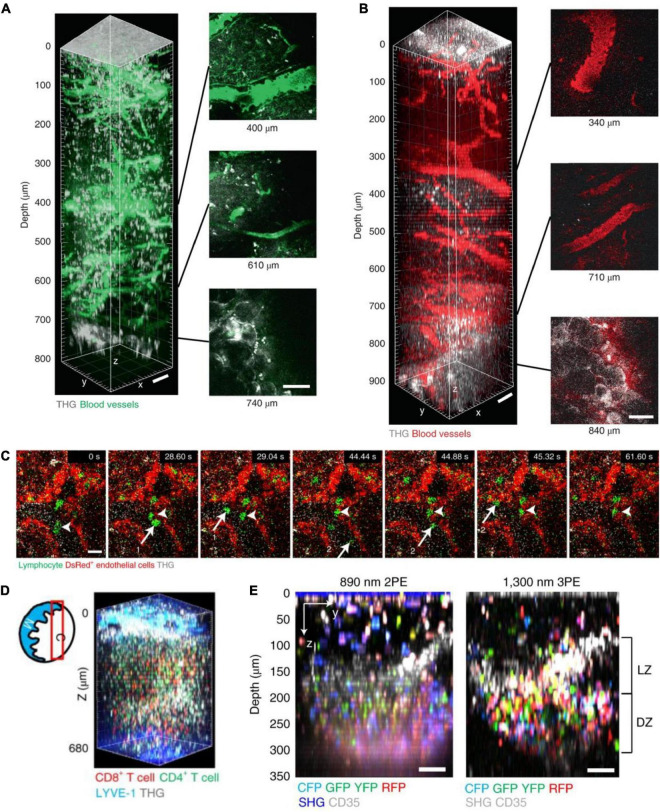
*In vivo* 3P imaging in mouse lymph nodes (LNs). **(A)** 3D reconstruction of 800 μm z-stack (left) and side views (right) taken with 1,280 nm 3PE, showing fluorescein-positive blood vessels and THG in a popliteal LN *in vivo*. Adipocytes were seen below the bottom of the lymph node at 740 μm depth in the THG channel. **(B)** 3D reconstruction of 900 μm z-stack (left) and side views (right) taken with 1,680 nm 3PE, showing Texas Red-positive blood vessels and THG in a popliteal LN *in vivo*. Adipocytes were seen below the bottom of the lymph node at 840 μm depth in the THG channel. Scale bars, 50 μm. **(C)** 3P time-series images depict lymphocytes (green, arrows 1 and 2) flowing in blood and lymphocytes (arrow heads) moving on the blood vessel wall (shown by DsRed^+^ endothelial cells, red) at 500 μm depth in popliteal LNs of actin–DsRed mice. Scale bar, 10 μm. **(D)** Diagram of imaging location (red box) in a LN; C, cortical side; M, medullary side in the LNs. 3D reconstruction of 620–680 μm z-stacks with the FOV of 404 μm× 404 μm taken with 1,300 nm 3PE, showing naive CD8^+^ T cells labeled with eGFP and naive CD4^+^ T cells labeled with DsRed. **(E)** Images of multicolor B cell in a GC of the popliteal LN of Cγ1Cre-confetti mouse were taken with 2PM and 3PM. Scale bars = 50 μm. LZ, light zone; DZ, dark zone. Adapted with permission from Choe et al. (2022).

A recent work applied 3PM imaging to visualize the entire popliteal lymph nodes (LNs) to explore the dynamic behavior of immune cells (Choe et al., 2022). In this work, they used 3PM to image the blood vessels labeled by fluorescein and Texas Red in the intravital adult mice, as shown in Figure 10. The maximum depths were 800 and 900 μm, respectively. The maximum average power under the objective lens was 72 and 21 mW for 1,280 and 1,680 nm excitation, respectively. Moreover, 3PM at 1,300 nm excitation enabled 3D-tracking of the eGFP-labeled lymphocyte migration in the LN parenchyma of actin-DsRed transgenic mice at a depth of 600 μm. A 3D volume (202 × 202 × 35 μm^3^) was acquired every 8.9 and 26.7 s. The authors also recorded T cell migration dynamics in the entire depth of popliteal LNs in steady state and under LPS-induced inflammation by labeling CD4^+^ and CD8^+^ T cells with eGFP (or CMRA) and DsRed (or CFSE). Besides, the work showed Cγ1Cre-Confetti mice to image multicolor B cell migration in germinal centers. 3PM is a very promising way to study how immune cells behave in deep tissues and organs other than the popliteal LNs, which cannot be reached by 2PM.

### 5.2. Other living samples

#### 5.2.1. *Drosophila*

Besides rodents, 3PM has also been applied to capture neural structure and activity in behaving flies (Tao et al., 2017; Hsu et al., 2019; Aragon et al., 2022). In 2017, a work tested 3PM imaging of brain structure and function in flies with intact cuticles without surgical operation (Tao et al., 2017). 3PM can visualize the entire mushroom body (MB) structure and finer neuronal compartments, such as the axon terminals of second-order olfactory neurons, the ciliated endings of the auditory chordotonal neurons, and individual cell bodies of the odorant receptor neurons. All the structures were clearly visible in transgenic flies expressing mCherry or jRCaMP1b. Then the researchers performed 3PM imaging for odor-evoked calcium activity in MB Kenyon cells at ∼70 μm depth. The cells express the red calcium sensor, jRCaMP1b, through using the OK107 driver line. The excitation wavelength used in this experiment was 1,700 nm. The power was less than 100 mW. The lateral and axial resolution were ∼0.8 and 3 μm, respectively.

More recently, a study demonstrates that standard 3PM outperforms 2PM in deeper brain regions, such as the central complex, especially when cellular and subcellular resolutions are necessary (Aragon et al., 2022). For structural imaging, when the cuticle was removed, 3P imaging depth was increased to ∼300 μm, reaching to the bottom of the fly brain. The researchers imaged the entire brain in a transgenic fly expressing membrane-targeted GFP pan neuronally. In the cuticle-intact imaging preparation, 3P imaging can clearly distinguish the deeper ellipsoid body ring from neurons expressing GFP in the central complex. In these experiments, the power used at 1,320 nm 3P excitation is less than 11 mW. 3P images were taken with a FOV of 270 μm× 270 μm (512 × 512 pixels/frame). The lateral resolution was ∼1.2 μm. For functional imaging, under cuticle-removed preparation, the depth limit for 3PM is down to ∼250 μm (compared to ∼120 μm in 2P). And the depth limit for 3P cuticle-intact functional imaging was ∼120 μm (compared to ∼65 μm in 2P). The researchers used 3PM to capture neural activity in response to electrical stimulation across different depths of the entire fly brain. In these experiments, the researchers recorded neural activity in transgenic flies expressing GCaMP6s pan-neuronally. The average laser power at 1,320 nm excitation is 4 mW. 3P activity was taken with an FOV of 200 × 100 μm^2^ (256 × 128 pixels/frame) and a 6.5 Hz frame rate. The researchers also used 3PM to record natural stimulus, odor-evoked responses of Kenyon cells in the fly brain through the intact cuticle. These deep imaging results showed that 3P imaging may enable structural and functional imaging through the entire depth of the fly brain *via* the intact cuticle with a compressing fly head.

#### 5.2.2. Zebrafish

A recent work has demonstrated that 3PM can be used to non-invasively image adult zebrafish’s structure and function (Chow et al., 2020). In this work, 3PM imaged the entire telencephalon and deep into the cerebellum and optic tectum in 3- to 7-month-old adult zebrafishes. In all three brain regions, calcium imaging depth is up to 750 μm below the surface of the head. For functional imaging in all three brain regions, the imaged neurons were labeled by nuclear localized GCaMP6s under the elavl3 promoter in transgenic adults. The images were acquired in a FOV of 200 μm× 200 μm (280 × 280 pixels/frame) and at a frame rate of 4.25 Hz. For structural imaging, the imaged glutamatergic neurons were labeled with DsRed, tdTomato, and GFP in transgenic adults. The excitation wavelengths of these experiments were 1,300 nm for green light-emitting fluorophores and 1,700 nm for red light-emitting fluorophores, respectively. The used power was below 90 mW. The 3P imaging technique allows the monitoring of structural and functional dynamics at a cellular resolution in an intact adult zebrafish.

In summary, 3PM enables unprecedented exploration of living organisms with low invasion. When 3PM is utilized to its full potential, a variety of real-world imaging tasks will become possible.

## 6. Future directions and conclusion

### 6.1. More accessible regions and cell types

**FIGURE 11:**
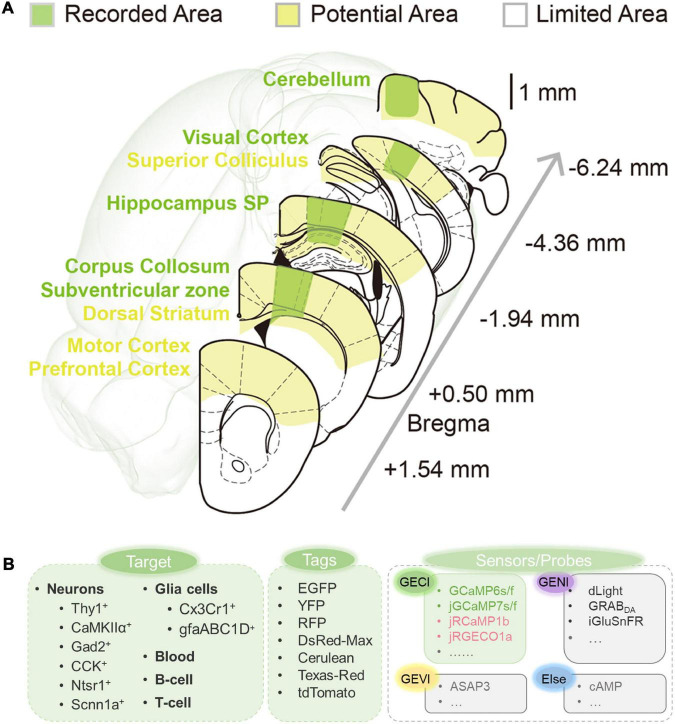
Summary of current and potential application 3PM. **(A)** Explored and potentially accessible regions in 3PM. Green shadows indicate areas that have been explored. Yellow shadows indicate potentially accessible areas. The white shadow denotes the regions beyond the present depth limitations and are inaccessible by surgery. **(B)** Fluorescent imaging application toolkit. Previous studies have explored the application of 3PM for various living components. A variety of fluorescence dyes and proteins have been analyzed. Several calcium indicators have been imaged through 3PM imaging, while various other types of sensors and probes through 3PM imaging have not been reported (indicated by gray).

As mentioned above, 3PM has now been successfully applied to structural and functional imaging in various living samples. Most of all, 3PM can image beyond the depth limitation of 2PM in the mouse brain (LaViolette and Xu, 2021). Many brain regions were successfully imaged while many are not yet: as shown in Figure 11A, yellow areas show potential areas in the near future, such as prefrontal cortex, dorsal striatum, and so on; and green areas show explored areas; and some white areas beneath the ears are hard for surgical operation in practical cases. It is promising to explore these accessible areas deep in the brain. From another perspective, until now, as shown in Figure 11B and Supplementary Table 1, various neural cells have been visualized by 3PM. Few reported works have achieved structural and functional imaging of transgenic or virally labeled mouse neurons including CaMKII-positive neurons and Thy1-positive neurons, GABAergic neurons (Gad2-positive neurons and CCK-positive neurons), and so on; and glia cells including astrocytes and microglia. In addition, dynamic imaging of T- and B-cell migration in the lymph nodes has also been achieved. Nevertheless, many more cell types, such as parvalbumin-positive interneurons, remain to be explored by 3PM in future studies. More species, such as monkeys, are also well worth exploring in the future.

### 6.2. More combinations with other technologies

In addition, the combination of 3PM and fluorescent technology yields various applications. One of the dispensable tools is the fluorescent indicator. A variety of fluorescent indicators are rapidly developing nowadays. Genetically encoded fluorescent probes are the best partners for optical imaging by virtue of their low invasiveness and long-term observation. The integration of 3PM and other technologies in neuroscience would also provide more solutions for many difficult scientific questions. For example, when combined with cue-related tasks and virtual reality systems, 3PM imaging could decode neuronal activities by behavioral information of the mice. And manipulation techniques such as optogenetics and chemogenetics will further enable the functional dissection of neural circuits.

## Author contributions

BL and YX conceived the idea for this review topics and organization. YX and PD performed the literature review and wrote the manuscript under the guidance of BL. All authors contributed to the article and approved the submitted version.
