# Supplementary material for: Three-photon excited fluorescence imaging in neuroscience: From principles to applications
Source: Front Neurosci. 2023 Feb 20;17:1085682. doi: 10.3389/fnins.2023.1085682 (PMC9986337; doi:10.3389/fnins.2023.1085682)
Supplement: Supplementary file 1 [file Table_1.DOCX]

**Supplementary Table. 1 The applications of *in vivo* 3PM imaging**

| Species | Animal | Region | Depth (μm) | | Sample type | | Label methods | Marker | Ref. | |
| --- | --- | --- | --- | --- | --- | --- | --- | --- | --- | --- |
| Mouse | FVB/N mouse |  | 1300 | | vasculature | | injection | Texas-Red–dextran | (Horton et al., 2013) | |
|  | Thy1-Brainbow1.0 |  | 1060-1120 | | pyramidal neuron | | transgenic mice | RFP |  |  |
|  | CaMKII-tTA/tetO-GCaMP6s | Hippocampal stratum pyramidale |  | | CaMKII^+^ neuron | | transgenic mice | GCaMP6s | (Li et al., 2020; Ouzounov et al., 2017; Wang et al., 2018) | |
|  | Thy1-jRGECO1a | Cortex | 750 | | Thy1^+^ neuron | | transgenic mice | jRGECO1a | (Li et al., 2020) | |
|  | Thy1-YFP-H | Cortex | 747-767 | | Thy1^+^ neuron | | transgenic mice | YFP | (Rodríguez et al., 2021) | |
|  |  | Hippocampus | 719 | | Thy1^+^ neuron | | transgenic mice | YFP |  |  |
|  |  | Dorsal horn of the spinal cord | 400 | | neuron | | AAV8-Syn-jGCaMP7s | jGCaMP7s |  |  |
|  | Thy1-EGFP(M) | Cortex | 1450 | | Thy1^+^ neuron | | transgenic mice | EGFP | (Streich et al., 2021) | |
|  |  | Hippocampus |  | | Thy1^+^ neuron | | transgenic mice | EGFP |  |  |
|  | Gad2-Cre::Ai14 | Hippocampus |  | | Gad2^+^ neuron | | transgenic mice | tdTomato |  |  |
|  |  | Corpus callosum | 1037 | | Fibrous astrocyte | | AAV2/5-GFAP-GCaMP6s | GCaMP6s |  |  |
|  |  | Corpus callosum | 862 | | Fibrous astrocytes | | AAV2/5-GFAP-GCaMP6s | GCaMP6s |  |  |
|  |  | Layer 6 of the visual cortex | 835 | | Protoplasmic astrocyte | | AAV2/5-GFAP-GCaMP6s | GCaMP6s |  |  |
|  |  | Cortex | 784 | | protoplasmic astrocyte | | AAV2/5-GFAP-GCaMP6f | GCaMP6f |  |  |
|  | CCK-Cre::Ai162D | Somatosensory cortex |  | | CCK^+^ neuron | | transgenic mice | GCaMP6s | (Qin et al., 2022) | |
|  | Scnn1a-Cre | Visual cortex | -- | | sodium channel, nonvoltage-gated 1 alpha^+^ neuron (cortex layer 4) | | AAV1/2-hSyn-FLEX-jGCaMP7f | jGCaMP7f | (Klioutchnikov et al., 2022) | |
|  | Ntsr1-cre | Visual cortex | -- | | neurotensin receptor 1^+^ neuron (cortex layer 6) | | AAV1/2-hSyn-FLEX-jGCaMP7f | jGCaMP7f |  |  |
|  | Cx3Cr1-GFP | cortex |  | | microglia | | transgenic mice | GFP |  |  |
|  | CD1 | White matter and SVZ | 1150–1472 | | RGC like neural Neural stem cell | | microinjection | CTO | (Sun et al., 2022) | |
|  |  | SVZ | 1386 | | Neuroblasts | | microinjection | CTO |  |  |
|  |  | OB | 1050 | | Neural stem cell | | microinjection | CTO |  |  |
|  |  | SVZ and LV |  | | Neural stem cell | | microinjection | Cell Tracker Green |  |  |
|  | Nestin-CreER2::Ai9 | SVZ | 1123 | | Neural stem cell | | transgenic mice | tdTomato |  |  |
|  |  | Popliteal lymph nodes | 600–900 | | vasculature | | injection | fluorescein; Alexa Fluor 647 |  |  |
|  |  | LPS-induced inflamed LNs |  | | CD8^+^ T cell; CD4^+^ T cell | | injection | CMRA; CFSE | (Choe et al., 2022) | |
|  | Cγ1Cre-confetti mouse | Popliteal lymph nodes |  | | GC B cell | | transgenic mice | CFP; GFP; YFP; RFP |  | |
|  |  | Popliteal lymph nodes |  | | blood vessel | | injection | Alexa-Fluor-647 |  | |
| Rat |  |  |  | |  | |  |  |  | |
|  | Lister Hooded rats | posterior parietal cortex; visual cortex neurons | 1120 | | neuron | | AAV1/2-Syn-GCaMP6s-WPRE | GCaMP6s | (Klioutchnikov et al., 2020) | |
| Zebrafish |  |  |  |  | |  |  |  | |  |
|  | Tg(vglut2a:loxP-DsRed-loxP-GFP |  |  | |  | | transgenic animal | DsRed, GFP | (Chow et al., 2020) | |
|  | Tg(vglut2a:mtdtomato) |  |  | |  | | transgenic animal | tdTomato |  | |
|  | Tg(vglut2a:EGFP) | the stratum periventriculare in the optic tectum | 750 | | glutamatergic neuron | | transgenic animal | EGFP |  | |
|  |  | entire Telencephalon | 1,045 | | elavl3 promoter | | transgenic animal | GCaMP6s |  | |
|  |  | Cerebellar | 700–750 | | Purkinje and granule cell | | transgenic animal | GFP |  | |
|  | Tg (kdrl:EGFP) |  |  | | vascular epithelial cells | | transgenic animal | EGFP | (Chen et al., 2018) | |
| Drosophila |  |  |  | |  | |  |  |  | |
|  |  | Mushroom body |  | | Kenyon cell | | transgenic animal | mCherry or jRCaMP1b | (Aragon et al., 2022) | |
|  |  | Mushroom body |  | | Kenyon cell | | transgenic animal | GFP or GCaMP6s |  |  |
|  | GH146>GCaMP5 strain | Antenna lobe |  | | excitatory projection neurons | | transgenic animal | GCaMP5 | (Chen et al., 2018) | |
